# Contribution of immune cells to bone metastasis pathogenesis

**DOI:** 10.3389/fendo.2022.1019864

**Published:** 2022-09-29

**Authors:** Ningning He, Jingting Jiang

**Affiliations:** ^1^ Department of Tumor Biological Treatment, The Third Affiliated Hospital of Soochow University, Changzhou, China; ^2^ Department of Oncology, Yangzhou University, Yangzhou, China; ^3^ Department of Oncology, First People’s Hospital of Changzhou, Changzhou, China

**Keywords:** immune system, immunotherapy, bone microenvironment, immune response, bone metastasis

## Abstract

Bone metastasis is closely related to the survival rate of cancer patients and reduces their quality of life. The bone marrow microenvironment contains a complex immune cell component with a local microenvironment that is conducive to tumor formation and growth. In this unique immune environment, a variety of immune cells, including T cells, natural killer cells, macrophages, dendritic cells, and myeloid-derived suppressor cells, participate in the process of bone metastasis. In this review, we will introduce the interactions between immune cells and cancer cells in the bone microenvironment, obtain the details of their contributions to the implications of bone metastasis, and discuss immunotherapeutic strategies targeting immune cells in cancer patients with bone metastasis.

## Introduction

**Figure 1:**
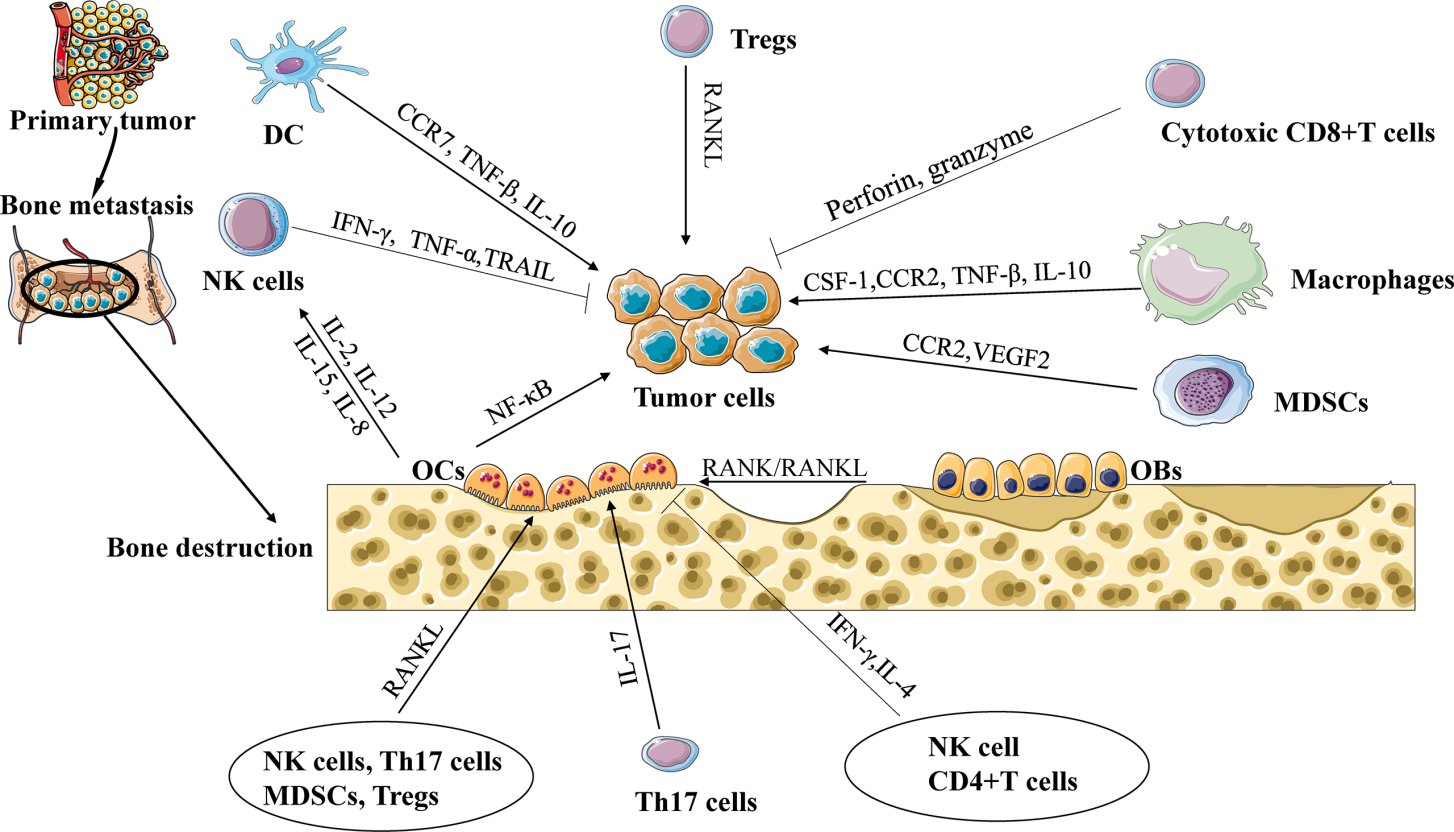
The interaction of immune cells, tumor cells, and osteocytes during bone metastasis. Cytotoxic CD8+ T cells release TNF-α and IFN-γ to eliminate tumor cells. Regulatory T cells (Tregs) promote tumor cell to bone metastasis through the RANK/RANKL axis. Tumor-associated macrophages (TAMs) promote tumor cell to bone metastasis through CCL2/CCR2 or CSF-1/ CSF-1R signaling. Natural killer cells (NK cells) can destroy tumor cells by secreting TNF-α and tumor necrosis factor-related apoptosis-inducing ligand. Dendritic cells (DCs) and TAMs suppress the cytotoxic capacity of CD8+ T cells via TGF-β, and interleukin-10 (IL-10) to promote tumor progression. Myeloid-derived suppressor cells (MDSCs) release chemokines, including vascular endothelial growth factor (VEGF) and CCL2/CCR2 signaling, to promote cancer progression and bone metastasis. IL-17 can also increase bone metastases, while IFN-γ and IL-4 secreted from Th1 and Th2 cells, respectively, can inhibit osteoclast formation and limit bone metastases. NK cells and CD4+ T cells support or repress the production of OCs controlled by the release of TNF-α or IFN-γ.

With the rise in morbidity due to cancer, bone metastasis has become the main reason for the death rate of people affected by carcinoma. The bone is one of the most important pathological process organs for various solid neoplasms, such as breast, lung, and prostate cancer (1). Although improvements have been made in the diagnosis and therapy of neoplasms, bone metastasis remains insurmountable. The formation and evolution of bone metastasis include involved communication occurring among tumor cells, immune cells, and osteocytes (2). In the spinal marrow, osteoblasts or osteoclasts release numerous growth factors that boost the expansion of metastatic tumors, leading to incurable osteoblastic or osteolytic lesions (3). The immune system is the primary defense system against tumor cells, and its effects on spinal metastasis are still unknown. Earlier studies concentrated on the interaction between tumor cells and bone progenitor cells, and recapitulating specific tumor cell–bone microenvironment interactions is lacking in *in-vivo* models. However, increasing evidence indicates that metastasis might rely on uncommon constraints in the tumor microenvironment (4). The antitumor or protumor impact of the immune microenvironment might rely on the existence of the regional cytokine milieu, tumor-specific interplay, and specific types of immune cells (**Figure 1**). Within the existing review, the elaborate functions and impacts of different immune cells on bone metastasis will be introduced. In addition, the existing therapeutic methods for bone metastasis will be presented.

## Interaction of tumor cells and immune cells in the bone microenvironment

The onset of neoplasm invasion of the bone leads to decoupled bone loss and formation, an essential process elicited by tumor cells and directed by osteoblasts (OBs) and osteoclasts (OCs) (5). Osteoblasts and osteoclasts are two major cell varieties in the bone microenvironment that promote bone metastasis. Tumor cells release signaling molecules that promote the differentiation of OCs and OBs (6), thus establishing the regeneration of the resultant tumor adhesion, proliferation, and increased bone mass (7). Disseminated tumor cells (DTCs) need to escape immune tolerance by migrating from the primary tumor to the bone. Once DTCs enter the bone marrow, they will proliferate or go dormant (8). In fact, whether DTCs escape from a dormant state is determined by factors released by the bone microenvironment (9), physical factors (10), and the activity of OBs and OCs (11). Evidence indicates that the stem cell released by OCs might trigger the nuclear factor-κB (NF-κB) pathway to induce tumor cell responses (12). Interleukin (IL)-6 plays a functional role in mediating the crosstalk between primary tumors and the bone marrow to promote monocyte-dendritic progenitors to give rise to immunosuppressive macrophages which, in turn, promote metastasis *in vivo* (13). An elementary molecule connected with the immune system and bone is supported by the specific axis involving RANKL, RANKL, and osteoprotegerin (14). This interaction of RANK/RANKL provokes osteoclast generation, whereas osteoprotegerin (OPG) offsets this impact by interacting with RANK (15). There is proof that interferon-γ (IFN-γ) created by stimulated CD4^+^ T cells might repress the common activity of osteoclasts (16). In contrast, activated regulatory T cells (Tregs) and type 17 T helper cells (Th17 cells) induce a protumor effect *via* immune repressions and osteoclast differentiation *via* RANKL (17).

## Effect of immune cells on bone metastasis

### T cells

T cells are derived from hematopoietic stem cells and lymphoid precursors kept in the spinal marrow. The thymus is the place where T cells experience early differentiation and are then kept in secondary lymphoid organs, where they are aroused by antigen presentation. Classical T helper 1 (Th1), 2 (Th2), or 17 (Th17) cells or induced T regulatory cells (Tregs) are distinct subsets polarized by naive CD4^+^ T cells (Th0), as demonstrated by the cytokines they will be recruited to (18). In CD4^+^ T cells, as latent immune inhibitors, Tregs play a vital role in the balance of the immune system (19). Interestingly, a salient increase in Tregs in the spinal marrow was observed following potential contact with metastatic prostate cancer, which is perceived to inhibit osteoclast formation and bone resorption (20). In addition to having immunological disorder functions, FOXP3^+^ Tregs are indisputably a significant source of RANKL (21). RANKL is the vital cytokine needed for osteoclast differentiation and tumor cell migrating to the bone (22), indicating that RANKL^+^ Tregs might stimulate DTC recruitment. Evidence suggests that tumor-specific Th17 cells can promote osteoclast activation and produce RANKL to induce osteolytic bone lesions (17). IL-17 can also increase bone metastases, while IFN-γ and IL-4 secreted from Th1 and Th2 cells, respectively, can inhibit osteoclast formation and limit bone metastases (23). CD8^+^ T cells can destroy tumor cells by secreting cytotoxic proteins (perforin and granzyme) through the Fas–Fas ligand axis mediating apoptosis (4). Furthermore, non-activated T cells increase osteoclastogenesis, whereas activated T cells are essential effectors in the protective impact against skeletal metastasis (24, 25).

The spinal marrow is a repository for the recruitment of memory CD4^+^ T cells (26) and CD8^+^ T cells (27), in which bone-derived cytokines, such as IL-7, influence them. Under the control of IL-7, the bone marrow induce the differentiation of CD4+ and CD8+ T cells from effector to memory T cells. (28). Feuerer et al. suggested that the number of memory CD4^+^ and CD8^+^ T cells in the spinal marrow of people with breast neoplasms is increased compared with that in healthy individuals (29).

### Macrophages

Macrophages are derived from spinal marrow progenitor cells derived from the yolk sac (30). Similarly, polarized macrophages with a pro-remodeling M2 or pro-inflammatory M1 phenotype were assessed (31). Inflammatory macrophages are attracted to the tumor microenvironment, are referred to as tumor-associated macrophages (TAMs), and are related to unfavorable prognoses in solid cancers (32). T-cell immunoglobulin and mucin domain-containing protein 4 (Tim4^+^) on TAMs could trap and inhibit CD8^+^ T-cell cytotoxicity and proliferation for promoting metastasis (33, 34). Macrophages are a major component in the tumor microenvironment arising from spinal marrow-derived monocyte differentiation in response to CC chemokine 2 (CCL2/CCR2) (35) and colony-stimulating factor 1 (CSF-1/CSF-1R). Furthermore, CSF-1 has been verified to be involved in macrophage-driven bone metastasis (36). Cellular adhesion and motility in macrophages are regulated by CCL2–CCR2 signaling (37). Breast tumor cells expressing CCL2 bind to monocyte-derived CCR2^+^ stromal cells, including macrophages and preosteoclast cells, to promote colonization in the lungs and bone (38). In prostate cancer, TAMs promote the invasion of tumor cells *via* CCL2–CCR2 signaling (39). In addition, recent studies have demonstrated that bone tumor growth can be weakened by the repression of macrophage-recruiting factors and TAM reprogramming from M2 to M1 (40). TAM-derived transforming growth factor-β (TGF-β) could facilitate the invasion of colorectal cancer cells (41). On the other hand, the final TAM molecular theories in the impetus of skeletal metastasis have not yet been represented. Simultaneously, another type of macrophage, called metastatic-associated macrophages (MAMs), is vital to motivate the generation of growth factors and inhibit T-cell antitumor responses (42).

### NK cells

NK cells experience upgrowth and become divided from CD34^+^ progenitors in the spinal marrow. The FcγIII^−^ receptor (CD16) is expressed by most NK cells, which pushes NK cells to mediate antibody-dependent cellular cytotoxicity (ADCC). NK cells can destroy tumor cells by secreting TNF-α and tumor necrosis factor-related apoptosis-inducing ligand (43), or cytokines, which are capable of reducing tumor cell proliferation and accelerating the inflammatory response, such as IFN-γ. In addition, NK cells are able to secrete chemokines to attract T cells, dendritic cells, and monocytes (44), leading to a specific crosstalk in the adaptive antitumor response (45). Leukemia cells can inhibit NK cells *via* RANKL signaling (46). However, OCs can be stimulated by NK cells *via* motivating the RANKL pathway under inflammatory conditions (47). NK cells support or repress the production of OCs controlled by the release of TNF-α or IFN-γ, respectively (48). Furthermore, NK cells appear to produce IFN-γ in response to a target combination of foreign cytokines such as IL-2, IL-12, IL-15, and IL-18.

### Dendritic cells

DCs originate from common myeloid progenitors (CMPs), which differentiate into common dendritic cell progenitors (CDPs) in the absence of the transcription factor Nur77, resulting in the generation of plasmacytoid DCs (pDCs) and conventional DCs (cDCs) (49). The transportation of cDCs, which are divided into cDC1 and cDC2, to lymph nodes and the production of a systemic antineoplastic immune response are controlled by C-C chemokine receptor 7 (CCR7) expression (50). The cDC1 subset has the unique capability to cross-present a foreign antigen, thereby activating Foxp3^+^ CD8^+^ Tregs (51, 52). cDC2s are responsible for presenting foreign antigens to CD4^+^ T cells and shaping the polarization of cells (53). Essentially, a consanguineous correlation between cDC2 abundance and non-Treg CD4^+^ T-cell infiltration into head and neck squamous carcinomas is demonstrated. For example, longer progression-free survival was associated with supernal cDC2s and poor Treg infiltration (54). pDCs seem to be primarily tolerated in the background of cancer and are associated with a negative prognosis (55). The upregulation of MHC molecules and costimulatory molecules is induced by enabled pDCs, which can still activate CD4^+^ and CD8^+^ T cells. It has also been found that tumor-infiltrating DCs and TAMs suppress the cytotoxic capacity of CD8^+^ T cells *via* the production of TGF-β and IL-10 (2). Similarly, pDCs recruit other immunonegative immune cells involving Tregs, and myeloid-derived suppressor cells (MDSCs) promote, but do not protect, tumor progression and metastasis (56). Subsequently, Sawant et al. reported an increasing number of pDCs in the spinal marrow of mice inoculated with breast 4T1 carcinoma cells (57); thus, therapeutically targeting pDCs might hold promise for treating bone metastasis.

### MDSCs

As a heterogeneous group of immature myeloid cells, MDSCs are derived from the spinal marrow. MDSCs are made up of two large groups: granulocytic or polymorphonuclear MDSCs (PMN-MDSCs) and monocytic MDSCs (M-MDSCs). Importantly, breast, ovarian, and gastric human neoplasms cultured *in vitro* secrete CCL2, and MDSCs from these patients express the relevant CCR2 and migrate toward these chemokines *in vitro* (58). Deletion of CCL2 in a mouse model of spontaneous colorectal neoplasm diminished the number of colonic MDSCs (59). PD-L1^+^ M-MDSCs can differentiate into osteoclasts and are potent suppressors of T-cell activation (25). In addition to effector T-cell groups, these data suggest that MDSCs might affect the expansion and activation of Tregs and conversely mediate immunosuppression. MDSCs from the bone microenvironment with bone metastases can subsequently differentiate into functional osteoclasts, and without bone metastases, they fail to differentiate into osteoclasts, which illustrates that tumor cells residing in the bone microenvironment lead to an increased quantity of activated osteoclasts (60, 61). Enhanced levels of MDSCs were found in the blood of patients with breast (62) and prostate neoplasms (63), which is correlated with the tumor stage. The higher the level of circulating MDSCs in patients with breast and prostate cancer, the lower the overall survival (64). Considering the potent impacts of MDSCs on destroying host immunity and quickening bone damage, MDSCs might become a latent therapeutic target for bone metastasis.

## Potential of modulating the immune system in the treatment of bone metastasis

### Targeting T cells

T cells can express receptors, such as cytotoxic T lymphocyte-associated protein 4 (CTLA-4) or apoptotic process protein 1 (PD-1), and when they interact with ligands, T cells lose activity. The monoclonal antibodies that intercept CTLA-4, PD-1, or PD-L1 show significant clinical results in patients with multiple neoplasms involving advanced melanoma (65) and non-small cell lung neoplasms (66). The anti-CTLA-4 antagonists and anti-PD-1 antibody nivolumab can block the inhibitory function of Tregs *in vitro*, as demonstrated in mice (67). Tim-4 inhibition significantly improves antitumor effectiveness in mouse models of anti-PD-1 treatment (34). Emerging data suggest that the androgen receptor is a negative regulator of CD8^+^ T cells in responding to anti-PD-1/PD-L1 treatment (68). Moreover, the usage of sunitinib and sorafenib which aims at VEGFR2 decreases the percentage of Tregs in foreign blood (69). To boost the immune response against tumors, CD8^+^ T cells are stimulated by vaccination or engineering T cells to express tumor-specific T-cell receptors (TCRs) or chimeric antigen receptors (CARs) (4). Engineered T cells were still detectable 9 months after transplantation in three patients in a phase I trial, and the number of cancer cells in two patients with refractory advanced myeloma was reduced. This result highlights the feasibility and therapeutic capacity of engineering cancer-specific T cells to attack cancer in the bone microenvironment (70). Clinical studies have not shown whether bone metastases can be reduced or eliminated by engineered T cells.

### Targeting macrophages

In view of the crucial character of macrophages in influencing bone metastasis, targeting macrophages would be an essential approach for skeletal metastasis therapy. Several therapeutic antibodies and molecules alone or in combination with other treatments are used to target TAMs. These treatments include depletion, reprogramming, and molecular targeting.

Inhibiting CSF-1/CSF-1R signaling and using liposomes containing clodronate are the most studied therapeutic approaches to remove TAMs from the tumor microenvironment. Anti-CD115 antibody (CSF-1R antibody) treatment reduced the number of TAMs and bone destruction in a breast neoplasm mouse model (71). TGF-β1 and VEGFA in tumor cells were downregulated when we depleted macrophages in squamous cell carcinoma models (72), which demonstrated that VEGFA-dependent angiogenesis was reduced after TAM ablation.

Overwhelming studies have suggested that targeting the NF-κB/CCL2 signal might be beneficial for blocking TAM recruitment (73). In agreement with this, the use of celecoxib to suppress NF-κB and the downregulation of CCL2 attenuated TAM recruitment and increased the apoptosis of tumor cells in malignant glioma (74).

Oligonucleotide delivery technology is another common method to reshape TAMs, involving charge-altering released transporters and other nanoparticles. To restore the negative effects of TAMs, sunitinib and sorafenib aimed to limit STAT3 or STAT6 in macrophages, subsequently distorting macrophage polarization (75, 76).

### Targeting NK cells

The selection of NK-cell sources and the means of enhancing NK-cell function *in vivo* are the key factors influencing NK-cell therapy. IL-2 and IL-15 are viewed as essential cytokines that upregulate the viability of NK cells. Treating human foreign blood mononuclear cells with IL-2 results in the expansion of a group of lymphokine-activated killer (LAK) cells, which consist primarily of T cells and NK cells and are highly cytotoxic to tumor cells (77). Furthermore, IL-2 decreased the amount and size of metastases in mouse models of pulmonary osteosarcoma when injected repeatedly at low doses following adoptive LAK cell transfer (78). In initial studies in syngeneic mouse models of several neoplasms, recombinant IL-15 was well tolerated and expanded NK and CD8^+^ T-cell groups, which promoted tumor suppression and reduced metastasis (79).

### Targeting dendritic cells

The capability of DCs to elicit robust and direct adaptive immune responses has been exploited for neoplasm immunotherapy, and targeting DCs may provide a way to improve immune responses. There is evidence that antibodies against vascular endothelial growth factor enhance antitumor immune responses by offsetting DC suppression (80, 81).

DC vaccination is the injection of mature DCs loaded with tumor antigens *ex vivo* into cancer patients. Whether this is clinically feasible has not been established, especially given the lack of circulating mature cDC1s in human foreign blood (55, 82). In glioblastoma, a phase III trial (NCT00045968) will evaluate the efficacy of a whole-cell DC vaccine unified with tumor resection, temozolomide, and radiotherapy, which showed safety and potential efficacy in earlier results (83).

Unlike injecting exogenously expanded and activated cDCs, injecting an incremental number of cDCs within tumors is another method to increase the cumulative function of the group. Preclinical studies have shown that systemic injection of Flt3L results in systemic expansion of the cDC1 population, enhances the number of these cells within B16 melanomas, and prominently destroys tumor growth (84). The anti-CD123-directed diphtheria toxin tagraxofusp-erzs was able to eliminate the pDC population in acute myeloid leukemia (85). This means that it is being studied clinically in several types of tumors, including metastatic breast cancer and non-Hodgkin’s lymphoma (NCT03789097, NCT01976585).

### Targeting myeloid-derived suppressor cells

Several lines of evidence manifest a close connection between MDSC accumulation and clinical outcome in cancer patients (86). The frequency of M-MDSCs is conversely interrelated with the treatment effect of chemotherapy in cervical and colorectal neoplasms (87, 88). The number of PMN-MDSCs is negatively correlated with the response to chemotherapy in colorectal cancer (88). In patients with unresectable melanoma, the percentages of circulating M-MDSCs and PMN-MDSCs are inversely associated with objective clinical responses to ipilimumab (anti-CTLA-4) (89, 90). Recent studies in mouse tumor models indicate that inhibition of MDSCs during immunotherapy improves the treatment effect (91, 92). Lu et al. supplemented a combination of low-dose adjuvant epigenetic modifiers in a mouse model of lung metastasis, which disrupts the formation of the pre-metastatic microenvironment by suppressing the migration of MDSCs and promoting MDSC differentiation into an interstitial macrophage-like phenotype (86). Fewer circulating MDSCs with lower iNOS and arginase expression and a greater number of spontaneously generated tumor-specific T cells are found in head and neck cancer and multiple myeloma patients who are treated with tadalafil (90, 93).

## Conclusions

In conclusion, the complex interactions among tumor cells, immune cells, and osteocytes in the spinal marrow microenvironment demand further research into the mechanisms that trigger bone metastasis. The function and number of immune cells in the bone microenvironment influence the efficacy of the anticancer immune response (**Table 1**). A comprehensive understanding of the roles and functions of T cells, macrophages, NK cells, DCs, and MDSCs in the bone microenvironment is essential for more effective treatments for bone metastasis and brings new promise to patients with bone metastasis.

**Table 1:** The interaction of immune cells, tumor cells, and osteoclasts in the bone microenvironment and their therapeutic strategies.

Immune cells	Tumor cells	Osteoclasts	Therapeutic strategies	References
CD8^+^ T cells	Perforin, granzyme	RANKL	Vaccination or engineering T cells to express tumor-specific T-cell receptors or chimeric antigen receptors	(4)
Tregs	RANKL	RANKL	Anti-CTLA-4 antagonists, anti-PD-1 antibodies nivolumab, sunitinib, and sorafenib	(65–69)
Macrophages	CSF-1, CCR2	–	Depletion by CSF-1 inhibitors or CCL2 inhibitors, reprogramming by sunitinib and sorafenib, molecular targeting	(71–76)
NK cells	IFN-γ, TNF-α, TRAIL	IFN-γ, IL-4	IL-2 and IL-15 are essential cytokines that upregulate the liveness of NK cells	(77–79)
Dendritic cells	CCR7, TNF-β	–	VEGF inhibits DC maturation and DC vaccination and increases the number of intratumoral cDCs	(81–85)
MDSCs	CCR2, VEGF2	RANKL	Targeting of MDSCs by chemotherapy, ipilimumab (anti-CTLA-4), and the PDE-5 inhibitor tadalafil	(87–93)

## Author contributions

The manuscript was conceptualized by NH and JJ. NH wrote the majority of the manuscript and produced the figure and table. JJ critically revised the manuscript. All authors contributed to the article and approved the submitted version.

## Funding

This work was supported by the National Key R&D Program (grant number 2018YFC1313400); the Joint Research Fund for Overseas Chinese, Hong Kong and Macao Scholars (grant number 31729001); and the National Natural Science Foundation of China (grant numbers 81972869, 81902386).

## Conflict of interest

The authors declare that the research was conducted in the absence of any commercial or financial relationships that could be construed as a potential conflict of interest.

## Publisher’s note

All claims expressed in this article are solely those of the authors and do not necessarily represent those of their affiliated organizations, or those of the publisher, the editors and the reviewers. Any product that may be evaluated in this article, or claim that may be made by its manufacturer, is not guaranteed or endorsed by the publisher.
